# Obesity reduces left ventricular strains, torsion and synchrony in mouse models: a cine DENSE MRI study

**DOI:** 10.1186/1532-429X-15-S1-P155

**Published:** 2013-01-30

**Authors:** Sage P Kramer, David Powell, Cassi Binkley, Lisa Cassis, Frederick H Epstein, Brandon K Fornwalt

**Affiliations:** 1 Pediatrics, Cardiology, and Physiology, University of Kentucky College of Medicine, Lexington, KY, USA; 2 Center for Biomedical Engineering, University of Kentucky, Lexington, KY, USA; 3 Graduate Center for Nutritional Sciences, University of Kentucky College of Medicine, Lexington, KY, USA; 4 Biomedical Engineering, University of Virginia, Charlottesville, VA, USA

## Background

Obesity is an epidemic affecting over 1 in 3 adults in the United States. Patients who are obese have increased cardiovascular mortality compared to those with normal weight, partly due to direct effects on the heart. However, the effect of obesity on advanced measures of cardiac function such as strain, torsion and synchrony are poorly understood.

## Methods

Ten 12-week-old male C57BL/6 mice were randomized to one of two diets: 1) a high-fat ‘western' diet with 60% of calories from fat or 2) a low-fat diet with 10% of calories from fat. After 5 months on the diet, the mice were imaged on a 7T ClinScan MRI (Bruker, Ettlingen, Germany) using a cine DENSE protocol and a 4-element phased array cardiac coil. Three short-axis slices (basal, mid, apical) and two long-axis slices (4-chamber and 2-chamber) were acquired with 15-20 frames per cardiac cycle. These data were then used to quantify left ventricular strain, torsion and measures of synchrony using semi-automated post-processing software written in MATLAB (Mathworks, Natick, MA).

**Figure 1:**
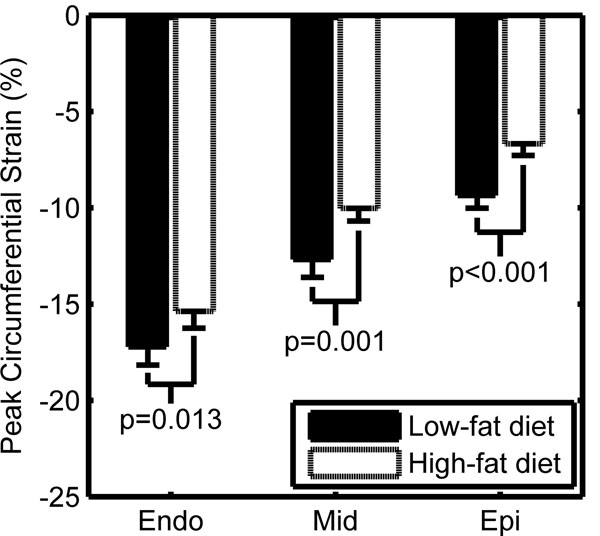
Obesity reduces left ventricular circumferential strains in mouse models.

## Results

Left ventricular sub-epicardial strain was significantly lower in the obese mice with a 40% reduction in circumferential strain (p<0.001), a 19% reduction in longitudinal strain (p=0.03), and a 53% reduction in radial strain (p=0.06). By contrast, left ventricular sub-endocardial strain was modestly reduced in the obese mice in the circumferential direction by 12% (p=0.01), and not significantly different in the radial (p=0.63) or longitudinal (p=0.44) directions. Peak left ventricular torsion was reduced by 34% in the obese mice (p=0.06). Finally, the radial uniformity of strain index showed a reduction in the synchrony of contraction in the left ventricle (p=0.01) with a time delay in the septal to free wall direction.

## Conclusions

Diet-induced obesity leads to a reduction in cardiac contractility in mouse models as evidenced by reductions in left ventricular strains and torsion. Reductions in cardiac strain are mostly limited to the sub-epicardial layer of the left ventricle, with relative preservation of function in the sub-endocardium. Diet-induced obesity also leads to reduced synchrony of contraction in the left ventricle of the heart.

## Funding

This work was supported by an NIH pilot project from a COBRE grant (5P20RR021954-05 and 8P20GM103527-05), the University of Kentucky Cardiovascular Research Center, and contributions made by local businesses and individuals through a partnership between Kentucky Children's Hospital and Children's Miracle network.

**Table 1:** 

	Region	Change due to high-fat diet	p
Radial Strain (%)	Endo	+5%	0.63
	
	Epi	-53%	0.06

Circumferential Strain (%)	Endo	-12%	0.01
	
	Epi	-40%	< 0.001

Longitudinal Strain (%)	Endo	-6%	0.44
	
	Epi	-19%	0.03

Radial Uniformity of Strain	Average	-5%	0.01

Peak Torsion (°/mm)	Total	-34%	0.06

